# Isolation and characterization of marine sponge–associated *Streptomyces* sp. NMF6 strain producing secondary metabolite(s) possessing antimicrobial, antioxidant, anticancer, and antiviral activities

**DOI:** 10.1186/s43141-021-00203-5

**Published:** 2021-07-15

**Authors:** Nayer Mohamed Fahmy, Asmaa Mohamed Abdel-Tawab

**Affiliations:** 1 Marine Microbiology Laboratory, National Institute of Oceanography and Fisheries, Cairo, Egypt; 2 Marine Biotechnology and Natural Products Laboratory, National Institute of Oceanography and Fisheries, Cairo, Egypt

**Keywords:** *Streptomyces*, Marine sponge, Antiviral, Anticancer, Antioxidant, Antimicrobial

## Abstract

**Background:**

Actinomycetes associated with marine sponge represent a promising source of bioactive compounds. Herein, we report the isolation, identification, and bioactivity evaluation of *Streptomyces* sp. NMF6 associated with the marine sponge *Diacarnus ardoukobae*.

**Results:**

Results showed that the strain belonged to the genus *Streptomyces*, and it was designated as *Streptomyces* sp. NMF6 with the GenBank accession number MW015111. Ethyl acetate (EtOAc) extract of the strain NMF6 demonstrated a promising antimicrobial activity against *Staphylococcus aureus*, *Enterococcus faecalis*, *Vibrio damsela*, and *Candida albicans* and a strong antioxidant activity, which were confirmed by DPPH, ferric-reducing power, and phosphomolybdenum assays; results are expressed as ascorbic acid equivalents. NMF6 extract also demonstrated cytotoxicity against breast cancer cell line (MCF-7), hepatocellular carcinoma cell line (Hep-G2), and human colon carcinoma cell line (HCT-116); the selectivity index values were < 2. The extract showed promising antiviral activity against HSV-1, CoxB4, and hepatitis A viruses at concentrations that were nontoxic to the host cells, with the selectivity index values being 13.25, 9.42, and 8.25, respectively. GC-MS analysis of the extract showed the presence of 20 compounds, with bis(2-ethylhexyl) phthalate being the major component (48%).

**Conclusions:**

Our study indicates that the marine sponge–associated *Streptomyces* sp. NMF6 strain is a potential source of bioactive compounds that could be developed into therapeutic agents.

## Background

Exploration of novel drugs is crucial to overcome the failure of the currently used medications. It is known that pathogenic bacteria have developed resistance to new generations of antibiotics, and some of them may tolerate all the known antibiotics and cause acute infections that necessitate more toxic and more expensive drugs [1]. Like bacteria, viruses mutate over time and develop resistance toward antiviral agents [2]. Cancer is still one of the most life-threatening diseases worldwide. An estimated 10 million cancer-related deaths among 18 million cases diagnosed with cancer were reported in 2018, which is expected to increase to 26.4 million annual cases worldwide, with 17 million deaths by the year 2030 [3, 4]. Oxidative stress is associated with the development of several pathologies such as cancer, Alzheimer’s disease, cardiovascular disease, early aging, ischemia, liver injury, arteriosclerosis, inflammation, skin damages, diabetes mellitus, and arthritis. The most currently used synthetic antioxidants such as tert-butylated hydroquinone (TBHQ), butylated hydro anisole (BHA), and butylated hydro toluene (BHT) are unsafe and could exert carcinogenic and toxic side effects [5, 6]. Hence, the search for bioactive compounds could lead to the discovery of novel compounds that could solve all these chemotherapy issues.

Natural products obtained from different sources (including microorganisms, animals, and plants) have provided important medicines for the treatment of diverse human and animal diseases and represent a potential source of more potent drugs to overcome the failure of conventional drugs [7, 8]. Microorganisms are more amenable for the large-scale production of bioactive compounds and would overcome the limitations of obtaining drugs by field-harvesting of large quantities of macroorganisms [9]. Till date, marine microorganisms could be considered as a relatively undervalued source for the discovery of bioactive compounds and could provide novel bioactive compounds in terms of structure and bioactivity [10, 11]. Among marine microbes, actinomycetes are excellent producers of bioactive compounds possessing different biological activities such as antitumor, antibiotic, immunosuppressive, antioxidant, antiviral, and enzyme inhibition properties [12].

The majority of actinomycetes recovered from marine sources have originated from marine sponges, with *Streptomyces* being the most abundant genus. Approximately 22% of all the bioactive compounds obtained from marine actinomycetes have been derived from sponge–associated species [13, 14]. Several studies have reported the isolation of various novel bioactive compounds from sponge–associated streptomycetes [15–20]. Therefore, we hypothesized that the marine sponge *Diacarnus ardoukobae* could host bioactive actinomycetes. In this study, we report the isolation, characterization, and bioactivity screening of *Streptomyces* sp. NMF6 isolated from *D. ardoukobae* collected from the mangrove ecosystem located 17 km south of Safaga at the Egyptian Red Sea coast. We evaluated the antimicrobial, antioxidant, anticancer, and antiviral activities of the ethyl acetate extract (EtOAc) prepared from *Streptomyces* sp. NMF6. We also determined the chemical constituents of the extract using GC-MS analysis.

## Methods

### Sample collection and isolation of actinomycetes

*D. ardoukobae* was collected in May 2019 from the mangrove ecosystem located 17 km south of Safaga (26° 36′ 53″ N and 34° 24′ 47.71″ E) and transferred to the laboratory in sterile plastic bags containing sea water within 3 h of collection. The sponge tissue was homogenized in sterile sea water using WiseTis Homogenizer HG 15D (witeg Labortechnik GmbH, Germany). The homogenate was plated on starch casein nitrate agar (SCNA) prepared using 50% sea water and supplemented with cycloheximide (50 mg/L) and nalidixic acid (25 mg/L) and incubated for 14 days at 30 °C. Actinomycetes colonies were purified by streaking on SCNA medium and maintained on slant cultures at 4 °C [21].

### Screening of actinomycetes for antimicrobial activity

The isolates were grown in Waksman’s broth—containing (g/L): glucose, 10; peptone, 5; beef extract, 5—for 14 days under shaking conditions (180 rpm) at 30 °C and tested for antimicrobial activity by the agar well diffusion method [22] against *Enterococcus faecalis* ATCC 29212, *Staphylococcus aureus* ATTC 25923, *Escherichia coli* ATCC 8739, *Pseudomonas aeruginosa* ATCC 4027, *Vibrio damsela*, *Candida albicans* ATCC 10231, *Fusarium* sp., *Rhizoctonia solani*, and *Aspergillus niger*. Thereafter, an isolate designated as NMF6 was selected for further characterization and bioactivity evaluation as it exhibited a broad-spectrum antimicrobial activity and produced a brown extracellular metabolite when cultured in Waksman’s broth.

### Phenotypic characterization of strain NMF6

The morphological, biochemical, and physiological characteristics of the strain NMF6 were extensively investigated. The strain was grown on different media and under different culture conditions, including different NaCl concentrations (1–10%, w/v), different pH values (1–10), and different temperatures (10–50 °C), after which the color of aerial and substrate mycelia, production of diffusible pigment, and utilization of carbon sources were analyzed as described by Shrilling and Gottlieb [23]. The morphology of spore chain and the spore surface texture were observed by scanning electron microscopy [24]. Gelatin liquefaction, nitrate reduction, and extracellular hydrolytic enzyme production (amylase, cellulase, protease, and lipase) were evaluated as described by Williams et al. [25]. The behavior of the strain toward 10 antibiotics was evaluated using the disk diffusion method [26].

### Molecular identification

Genomic DNA was isolated as described by Sambrook and Russell [27]. Polymerase chain reaction (PCR) was conducted to amplify the 16S rDNA gene of the strain NMF6 using universal primers p27F (5′-AGAGTTTGATCCTGGCTCAG-3′) and 1492R (5′-TACGGCTACCTTGTTACGACTT-3′). The PCR product was purified and sequenced, and the BLAST program (www.ncbi.nlm.nih.gov/blst) was used to evaluate the similarity. Multiple sequence alignment and phylogenetic tree construction were performed using the Mega-X software, version 10.1.7 [17, 28].

### Fermentation and extraction of secondary metabolites

The crude extract of the strain NMF6 was obtained by fermentation and extraction. Briefly, the starting inoculum was prepared by growing the strain in tryptone soya broth for 5 days under shaking conditions (180 rpm) at 30 °C. Then, 2% of the starting inoculum was used to inoculate Waksman’s broth prepared using 50% seawater, distributed into 1-L Erlenmeyer flasks (400 mL/flask), and incubated in a rotary shaker incubator (180 rpm) at 30 °C for 14 days. Cell-free supernatants were collected after centrifugation at 8000 rpm and extracted using equal volumes of ethyl acetate, and the organic phase was collected and evaporated using a rotary evaporator [29, 30]. Stock solutions of the crude extract in methanol and dimethyl sulfoxide (DMSO) were prepared separately and stored at 4 °C until used in different assays. The crude extract was screened for different biological activities, including antimicrobial, antioxidant, anticancer, and antiviral effects.

### Determination of antimicrobial activity

The antimicrobial potential of EtOAc was evaluated by the disk diffusion method. Briefly, sterile filter paper disks (6-mm diameter) were impregnated with 30 μL (containing 0.5 mg) of methanol stock solution of the crude extract; control disks were impregnated with 30 μL of methanol without the extract. The disks were maintained at room temperature until the complete evaporation of methanol and then placed on the surface of agar plates previously seeded, separately, with the test microorganisms. The plates were incubated under suitable conditions for each of the test microorganisms, and the diameter of the inhibition zone was measured. The minimum inhibitory concentration (MIC) was determined using the broth dilution method. Briefly, twofold serial dilutions (62.5-975 μg/mL) of NMF6 extract were prepared in Muller Hinton broth. Negative control which should be turbid was prepared with the solvent (DMSO) without the extract, and the uninoculated media was considered as positive control. All of the tubes were inoculated with bacterial suspensions (0.5 McFarland turbidity standards), incubated at 37 °C for 24 h. The growth was measured at 625 nm using the JENWAY 6800 spectrophotometer [31].

### Determination of antioxidant activity

Three different assays were used to evaluate the antioxidant potential of the crude extract of the strain NMF6. In all assays, all the experiments were conducted in triplicate with a sample dose of 25–150 μg, and a parallel experiment was conducted using ascorbic acid (2–15 μg) as positive control, after which an ascorbic acid standard curve was developed. The antioxidant capacities of the extract were calculated as vitamin C equivalent antioxidant capacity values.

### DPPH assay

The DPPH (1-diphenyl-2-picrylhydrazyl) radical-scavenging activity of NMF6 extract was evaluated as described by Kasangana et al. [32]. Equal volumes of the extract (25, 50, 75, 100, 125, and 150 μg/mL) or vitamin C (2–10 μg/mL) were mixed with a methanolic solution of DPPH (0.2 mM) and incubated in the dark. After 30 min, the absorbance values of all samples and the blank (containing pure methanol instead of sample) were measured at 517 nm using the JENWAY 6800 spectrophotometer. The absorbance response (y) of vitamin C (y = 7.4948x − 3.4408, R^2^ = 0.9976) and concentrations (0–10 μg/mL) was linear, and the results are expressed as ascorbic acid equivalent antioxidant capacity (AAEAC) values. All experiments were conducted in triplicate, and the DPPH scavenging activity and ascorbic acid equivalents were calculated using the following equations:

$$ \%\mathrm{DPPH}\ \mathrm{scavenging}\ \mathrm{activity}=\frac{Abs\  Blank- Abs\  sample}{Abs\  Blank}\times 100 $$

$$ \mathrm{Ascorbic}\ \mathrm{acid}\ \mathrm{equivalents}\ \left(\upmu \mathrm{g}\right)=\frac{Abs\  of\ sample+3.4408}{7.4948} $$

### Phosphomolybdenum assay

The total antioxidant activity of NMF6 extract was evaluated using the phosphomolybdenum method. Briefly, 2.7 mL of phosphomolybdenum reagent (28 mM sodium phosphate, 0.6 M sulfuric acid, and 4 mM ammonium molybdate) was mixed with 0.3 mL of the extract or vitamin C to obtain various final concentrations (25, 50, 75, 100, 125, and 150 μg/mL) and 2–10 μg/mL, respectively. The mixture was incubated at 95 °C for 90 min, and the absorbance was measured at 695 nm using the JENWAY 6800 spectrophotometer [32]. The absorbance response (y) of vitamin C (y = 0.097x − 0.0041, R^2^ = 0.9999) and concentrations (0–10 μg/mL) was linear. All experiments were conducted in triplicate, and the AAEAC values were calculated using the following equation:

$$ \mathrm{Ascorbic}\ \mathrm{acid}\ \mathrm{equivalents}\ \left(\upmu \mathrm{g}\right)=\frac{Abs\  of\ sample+0.0041}{0.097} $$

### Ferric-reducing power assay

The ferric-reducing power of NMF6 extract was evaluated as described by Aliyu et al. [33]. Briefly, 0.5 mL containing different doses of the extracts (25, 50, 75, 100, 125, and 150 μg) was mixed with 0.5 mL of phosphate buffer solution (200 mM, pH = 6.6) and 0.5 mL of 1% potassium ferricyanide [K_3_Fe (CN)_6_]. The mixture was incubated at 50 °C for 20 min, allowed to cool to room temperature, and mixed with 0.5 mL of 10% trichloroacetic acid. A volume of 0.5 mL of this mixture was mixed with 0.5 mL of distilled water and 0.1 mL of FeCl_3_ (0.1% solution) and allowed to stand for 10 min, after which the absorbance was measured at 700 nm using the JENWAY 6800 spectrophotometer. The absorbance response (y) of ascorbic acid (y = 0.0205x − 0.0055, R^2^ = 0.994) and concentrations (0–10 μg/mL) was linear, and the results were calculated as AAEAC values using the following equation:

$$ \mathrm{Ascorbic}\ \mathrm{acid}\ \mathrm{equivalents}\ \left(\upmu \mathrm{g}\right)=\frac{Abs\  of\ sample+0.0055}{0.0205} $$

### Determination of anticancer activity

The cytotoxic effect of NMF6 extract on three human cancer cell lines, viz., breast cancer cell line (MCF-7), hepatocellular carcinoma cell line (Hep-G2), and human colon carcinoma cell line (HCT-116), and normal human lung fibroblast cells (Wi-38) was evaluated using 3-(4,5-dimethylthiazol-2-yl)-2,5-diphenyltetrazolium bromide (MTT) assay. Cells were cultured in Dulbecco’s modified Eagle’s medium supplemented with 10% fetal bovine serum and 1% antibiotic solution (penicillin-G and streptomycin) in 96-well plates and incubated for 24 h at 37 °C and 5% CO_2_. The cells were washed twice and seeded in 0.1 mL of Roswell Park Memorial Institute (RPMI) 1640 medium containing different concentrations of the extract (15.62–500 μg/mL). A control experiment was conducted using only solvent (DMSO) without the sample. The plates were incubated at 37 °C for 72 h with 5% CO_2_ and then treated with 20 μL of MTT solution (5 mg/mL in PBS) and further incubated for 1–5 h. The medium was decanted, and the formazan, formed by metabolically active cells, was dissolved in 100 μL of DMSO. Absorbance was measured at 570 nm, and the background was subtracted at 620 nm [34–36]. The values of IC_50_ (concentration of compound required to inhibit cell viability by 50%) were calculated from the dose-dependent curve using the GraphPad InStat software. The selectivity index (SI) was calculated as the ratio of IC_50_ for normal cells to that of cancer cells. Variations in cellular morphology of treated and nontreated cells were visualized using a phase-contrast inverted microscope (Olympus, Japan) equipped with a digital camera and the Cell Sense software.

### Determination of antiviral activity

#### Cell line and viruses

The Vero cell line (ATCC: CCL-81) and herpes simplex virus (HSV-1), Coxsackie B4 virus (CoxB4), and hepatitis A virus (HAV) were used in this study. All viruses were obtained from the Microbiology Department, Faculty of Medicine for Girls, Al-Azhar University, Cairo.

#### Determination of cytotoxicity of NMF6 strain extract on Vero cells

The cytotoxic activity of the different concentrations (15.62, 31.25, 62.5, 125, 250, and 500 μg/mL) of NMF6 extract on viral host cells (Vero cells, ATCC CCL-81) was evaluated using MTT assay as described earlier. The maximum tolerated concentration, which did not cause toxicity or morphological alteration toward Vero cells, was determined, and the percentage of cytotoxicity was calculated as [(A − B)/A × 100], where A and B are the absorbance values of control and treated cells, respectively. The CC_50_ value (the concentration that caused 50% toxicity) was determined from the cell viability standard curve using the GraphPad Prism 5 software program [37, 38].

#### Antiviral assay

Antiviral activity was evaluated using MTT assay as described previously [38]. Briefly, the Vero cell culture (10,000 cells/200 μL of media) was dispensed into each well of a 96-well plate (three wells were left empty as blank control). The plate was incubated overnight at 37 °C under 5% CO_2_ to permit adherence of cells to the wells. Next, 100 μL of virus/sample suspension, prepared by mixing equal volumes of nonlethal concentrations (15.62, 7.8, and 3.9 μL/mL) of NMF6 extract and virus suspension and incubated for 1 h, was added to each well, shaken for 5 min at 150 rpm, and incubated at 37 °C for 24 h under 5% CO_2_ to permit the activity of the virus. Then, 20 μL of MTT solution (5 mg/mL in phosphate-buffered saline) was added to each well, mixed thoroughly by shaking for 5 min at 150 rpm, and incubated for 1–5 h to allow metabolism of MTT. The medium was poured off, and the plate was dried to remove any residue. The MTT metabolic product (formazan) was resuspended in 200 μL of DMSO and shaken well to ensure complete solubility. The optical density was determined at 560 nm, and the background was subtracted at 620 nm. The IC_50_ value (the concentration required to reduce the viral cytopathic effect to 50%) was estimated using the GraphPad Prism 5 software program. The SI value was calculated as the ratio of CC_50_ to IC_50_, and the antiviral activity was calculated as follows:

$$ \mathrm{Antiviral}\ \mathrm{activity}\ \left(\%\right)=\left(A-B\right)/\left(C-B\right)\times 100 $$

where A, B, and C are the absorbance values of sample, virus, and cell control, respectively.

#### GC-MS analysis

GC-MS analysis was conducted according to previously described methods [39]. Briefly, the Thermo Scientific Trace GC1310 gas chromatograph attached with a mass spectrometer was used to identify the individual components present in the EtOAc of strain NMF6. The instrument was coupled with the Agilent J&W DB-5 column, and the temperature was set at 40–280 °C. The sample was injected at 300 °C and eluted under low-pressure helium gas (1 mL/min). The mass spectra of the compounds were compared with Wiley Registry8e library.

#### Infrared spectra

The FT-IR spectra of NMF6 extract were recorded using a Bruker Tensor 27 FTIR spectrophotometer and the conventional KBr disk method. A total of 32 scans were collected at a spectral resolution of 4 cm^−1^.

## Results

### Isolation of sponge–associated actinomycetes

Eight isolates of actinomycetes were retrieved from the marine sponge *D. ardoukobae* collected from the mangrove ecosystem. All isolates exhibited colonial morphology of the genus *Streptomyces*. The isolates were screened for antimicrobial activity. Due to distinct antimicrobial activity, one isolate, designated as strain NMF6, was selected for further characterization and bioactivity evaluation.

### Morphological and biochemical characterization of strain NMF6

**Fig. 1:**
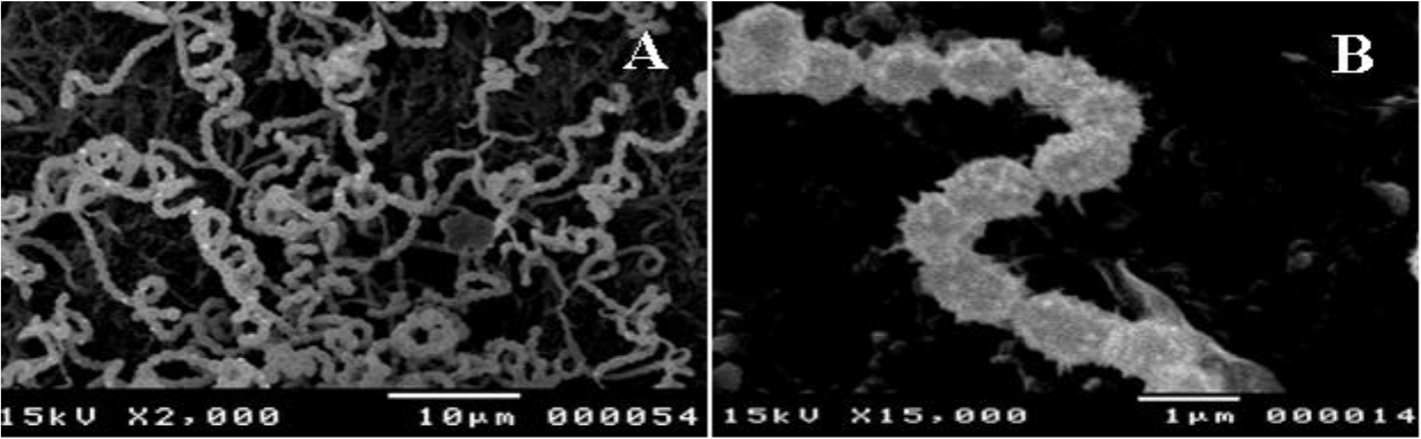
Scanning electron micrograph of NMF6 strain showing spiral spore chain (**A**) and spiny spore surface (**B**)

The strain NMF6 is gram-positive, aerobic, and filamentous with simple spiral spore chain and spiny spore surface. The strain grew well on all tested solid media with variable colony colors and produced a brown diffusible pigment on only Waksman’s medium. It produced catalase and hydrolyzed carboxymethyl cellulose, starch, gelatin, and Tween 80 but failed to reduce nitrate or hydrolyze urea. Among the carbon sources tested, the strain utilized only glucose, starch, and glycerol as the sole carbon source. It tolerated up to 4% of sodium chloride and grew well at a temperature range of 20–45 °C and a pH range of 4–10. The strain was resistant to clindamycin 5 μg, flucloxacillin 5 μg, ciprofoxacillin 5 μg, and trimethoprim/sulfamethoxazole 1.5/23.75 μg (Tables 1, 2 and Fig. 1).

**Table 1:** Morphological, biochemical, and physiological characteristics of NMF6 strain

Characteristic	Result
**Morphological characteristics**	
Gram	+
Spore chain	Spiral
Spore surface	Spiny
Diffusible pigment	+
**Physiological and biochemical properties**	
**Production of**	
Cellulase	+
Amylase	+
Lipase	+
Gelatinase	+
Urease	−
Catalase	+
Nitrate reduction	−
**Utilization of carbon sources**	
Glucose	+
Fructose	−
Maltose	−
Lactose	−
Arabinose	−
Xylose	−
Starch	+
Glycerol	+
Sucrose	−
Mannitol	−
**Resistance to**	
Amikacin 30 mcg	−
Flucloxacillin 5 mcg	+
Clindamycin 2 mcg	+
Erythrocin 15 mcg	−
Amoxicillin/clavulanic acid 20/10 mcg	+
Streptomycin 10 mcg	−
Ciprofoxacillin 5 mcg	+
Tobramycin 10 mcg	−
Trimethoprime/sulphamethoxazole 1.5/23.75 mcg	−
**Growth at different pH**	
4-10	+
**Growth at different NaCl (w/v, %**)	
0-4	+
**Growth at different temperature**	
20-45 °C	+
50 °C	−

**Table 2:** Cultural characteristics of strain NMF6 on various media at 30 °C for 14 days

Media	Growth	Colony color		Diffusible pigment
		Aerial mycelium	Substrate mycelium	
ISP1	Fastidious	Gray	Green	-
ISP2	Fastidious	Gray	Brown	-
ISP4	Fastidious	Gray	Green	-
ISP5	Fastidious	Gray	Yellow brown	-
ISP7	Fastidious	White	Yellow brown	-
SCNA	Fastidious	White	Yellow brown	-
WGA	Fastidious	Gray	Brown	Brown
NA	Fastidious	Gray	Green	-

*SCNA* starch casein nitrate agar, *WGA* Waksman’s glucose agar, *NA* Nutrient agar

### Molecular phylogeny of strain NMF6

**Fig. 2:**
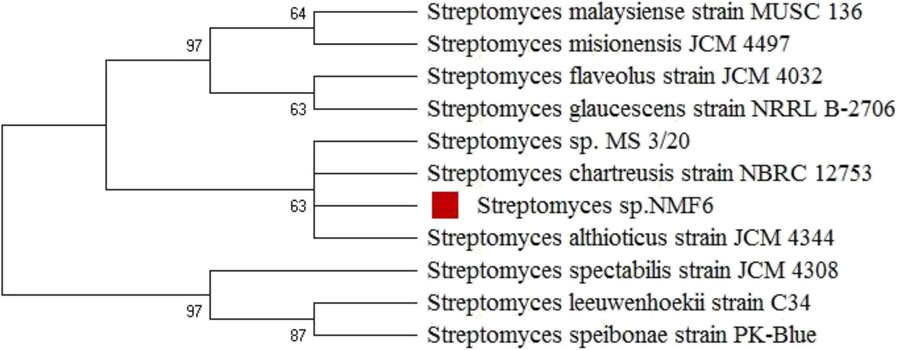
Neighbor-joining phylogenetic tree based on 16S rRNA sequences of NMF6 strain and related *Streptomyces* species. Numbers at nodes are bootstrap values (> 50%) based on 1000 resamplings

The partial 16S rDNA sequence of strain NMF6 was determined and compared with the sequences available in GenBank. The strain shared the highest similarity with 16S rDNA sequences of *Streptomyces* sp. MS 3/20, *S. chartreusis* strain NBRC 12753, and *S. althioticus* strain JCM 4344 (99.68%) and formed a single clade with these strains upon phylogenetic analysis based on the neighbor-joining method. The strain was deposited in GenBank as *Streptomyces* sp. NMF6 under the accession number MW015111 (Fig. 2).

### Antimicrobial activity of *Streptomyces* sp. NMF6

The antimicrobial activity of the EtOAc of strain NMF6 was evaluated using the disk diffusion method, and the MIC values were determined using the broth dilution method. The extract exhibited antimicrobial activity against *S. aureus*, *E. faecalis*, *V. damsela*, and *C. albicans* but failed to inhibit any of the tested filamentous fungi. The extract demonstrated the maximum antimicrobial activity against *V. damsela* followed by *E. faecalis* and *C. albicans*. *S. aureus* was the most resistant test microorganism and had the highest MIC value (Table 3).

**Table 3:** Antimicrobial activity and MIC (μg/mL) of *Streptomyces* sp. NMF6

Test microorganism	*Zone of inhibition (mm)	MIC (μg/mL)
*Staphylococcus aureus*	16 ± 1	520
*Candida albicans*	17 ± 1	490
*Vibrio damsela*	25.3 ± 1.5	335
*Enterococcus faecalis*	22.7 ± 1.5	365

*Values are mean ± SD (n = 3)

### Antioxidant activity

The antioxidant capacity of *Streptomyces* sp. NMF6 extract in a dose range of 25–150 μg was evaluated by different assays and expressed as ascorbic acid equivalents. The *Streptomyces* sp. NMF6 extract exhibited significant antioxidant activity over a dose range of 25–150 μg. The antioxidant activity of the extract in all assays was dose-dependent, and the ascorbic acid equivalent values were the highest in the DPPH assay followed by phosphomolybdenum and ferric-reducing power assays (Table 4).

**Table 4:** Antioxidant activities demonstrated by *Streptomyces* sp. NMF6 extract expressed as ascorbic acid equivalents

Sample mass (μg)	*Ascorbic acid equivalent (μg)		
	DPPH assay	Phosphomolybdenum assay	Ferric-reducing power assay
25	3.37 ± 0.28	2.36 ± 0.19	1.91 ± 0.14
50	5.84 ± 0.29	4.10 ± 0.04	2.79 ± 0.23
75	7.96 ± 0.10	5.28 ± 0.30	3.98 ± 0.22
100	9.63 ± 0.08	6.22 ± 0.02	5.13 ± 0.12
125	10.78 ± 0.05	7.32 ± 0.07	6.53 ± 0.12
150	11.83 ± 0.14	7.70 ± 0.03	7.67 ± 0.15

*Data are expressed as mean ± standard deviation (n = 3)

### Anticancer activity

**Fig. 3:**
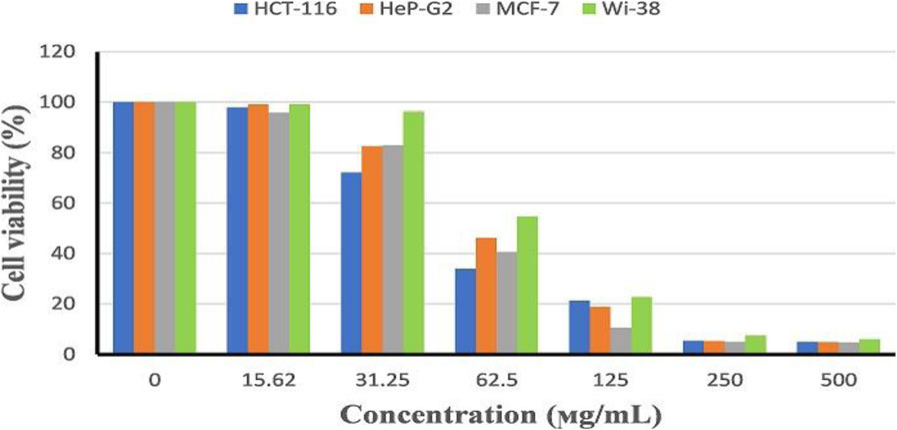
Cytotoxic activity of NMF6 extract against human breast cancer cell line (MCF-7), hepatocellular carcinoma cell line (Hep-G2), colon carcinoma cell line (HCT-116), and normal human lung fibroblast cells (Wi-38) as evaluated by MTT assay. Data are expressed as mean (n = 3) ± standard deviation

**Fig. 4:**
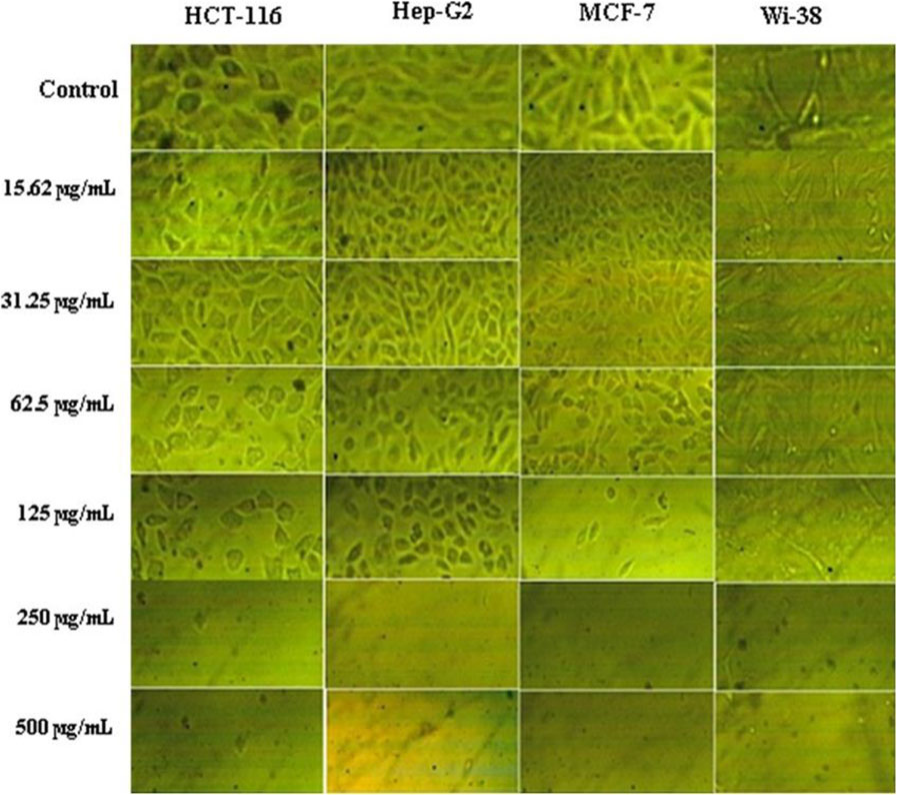
Cytotoxic effect and variation in morphological characteristics of human colon carcinoma cell line (HCT-116), hepatocellular carcinoma cell line (Hep-G2), breast cancer cell line (MCF-7), and normal human lung fibroblast cells (Wi-38) treated with different concentrations (15.62–500 μg/mL) of *Streptomyces* sp. NMF6 extract compared with untreated cell lines (control)

Figure 3 depicts the cytotoxic effect of *Streptomyces* sp. NMF6 extract toward three human cancer cell lines, viz., breast cancer cell line (MCF-7), hepatocellular carcinoma cell line, (Hep-G2), and human colon carcinoma cell line (HCT-116), and normal human lung fibroblast cells (Wi-38). The NMF6 extract markedly reduced the viability of all the tested human cancer cell lines at concentrations ranging from 15.62 to 500 μg/mL. The viabilities of the cancer cell lines at the maximum concentration of the extract (500 μg/mL) tested were 5%, 4.89%, and 4.84% for HCT-116, Hep-G2, and MCF-7, respectively. The normal human lung fibroblast cells (Wi-38) were less sensitive than the cancer cell lines; however; the SI values for the cancer cell lines were < 2 (Table 5). In addition to cell viability assessment using MTT assay, the effect of strain NMF6 extract on the appearance of cells was investigated. Treatment of cancer cells (HCT-116, Hep-G2, MCF-7) with NMF6 strain extract visibly altered the cell morphology. At concentrations ≤ 31.25 μg/mL, few apoptic bodies were observed while at concentrations (62.5-500 μg/mL) of the extract all cancer cells exhibited cytoplasm condensation, nuclear margination, and chromatin fragmentation. Normal human lung fibroblast cells (Wi-38) showed only cell shrinkage upon treatment with the extract (Fig. 4).

**Table 5:** IC_50_ and selectivity index of *Streptomyces* sp. NMF6 extract

Cell line	IC_50_	Selectivity index (SI)
		(IC_50_ of Wi-38 normal cell line/IC_50_ of cancer cell lines)
MCF-7	55.65	1.5
Hep-G2	59.39	1.4
HCT-116	49.85	1.67
Wi-38	83.56

### Antiviral activity

**Fig. 5:**
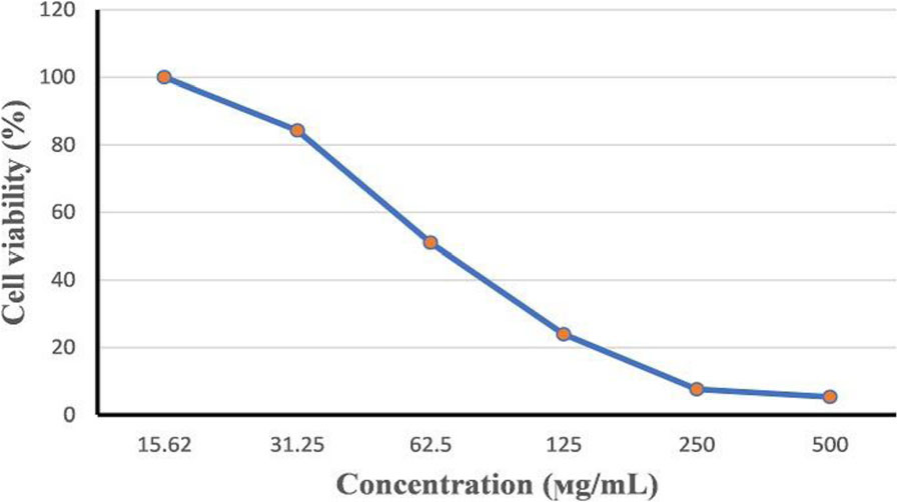
Cytotoxicity of NMF6 extract on Vero cells

**Fig. 6:**
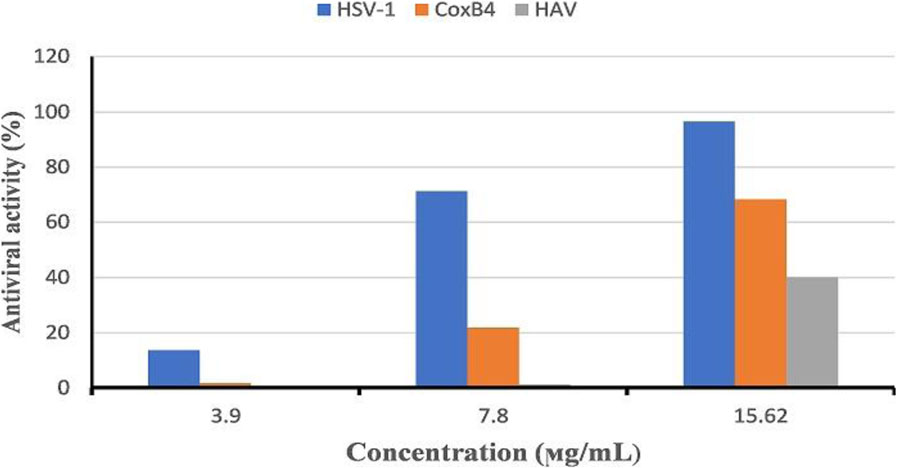
Antiviral activity of NMF6 extract against HSV-1, CoxB4, and HAV at concentrations nontoxic to Vero host cells

The maximum nontoxic concentration of NMF6 extract on Vero cells and the antiviral activity of concentrations equal to or below this concentration were evaluated using MTT assay. The 50% cytotoxic concentration (CC_50_) of the extract on Vero cells was 80.26 μg/mL, and the maximum nontoxic concentration was 15.62 μg/mL (Fig. 5). The antiviral activities of the extract at the maximum nontoxic concentration were 96.55%, 68.34%, and 40% against HSV-1, CoxB4, and HAV, respectively (Fig. 6). At the lowest tested concentration (3.9 μg/mL) of the extract, the antiviral activity was extremely weak against HAV and CoxB4, with the values being 0.15% and 1.68%, respectively (Fig. 6). The SI values, which were 13.25, 9.42, and 8.25 for HSV-1, CoxB4, and HAV, respectively, also confirmed these results (Table 6).

**Table 6:** Chemical compounds detected in the EtOAc of strain NMF6 by GC-MS analysis

RT	Compound name	M.W	M.F	Area %
14.54	Hydroquinone **(1)**	110	C_6_H_6_O_2_	2.61
14.91	1-Tetradecanol **(2)**	214	C_14_H_30_O	0.42
15.12	1-Hexadecanol **(3)**	242	C_16_H_34_O	1.50
16.30	2-Allyl-5-t-butylhydroquinone **(4)**	206	C_13_H_18_O_2_	0.64
16.63	Eicosane **(5)**	282	C_20_H_42_	0.79
18.09	Docosane **(6)**	310	C_22_H_46_	0.81
18.29	1-Nonadecene **(7)**	266	C_19_H_38_	4.74
18.91	1,2-Propanediol, 3-(octadecyloxy)-, diacetate **(8)**	428	C_25_H_48_O_5_	0.56
20.86	Methyl Nonadecanoate **(9)**	312	C_20_H_40_O_2_	0.66
21.18	1-Docosene **(10)**	308	C_22_H_44_	3.95
21.49	Methyl 14-methylpentadecanoate **(11)**	270	C_17_H_34_O_2_	1.50
22.95	Diisobutyl phthalate **(12)**	278	C_16_H_22_O_4_	1.43
23.55	7,9-Di-tert-butyl-1-oxaspiro (4,5) deca-6,9-Diene-2,8-dione **(13)**	276	C_17_H_24_O_3_	1.05
23.71	Erucic acid **(14)**	338	C_22_H_42_O_2_	0.58
23.83	1-Docosanol **(15)**	326	C_22_H_46_O	2.26
24.52	Phthalic acid, butyl 8-methylnonyl ester **(16)**	362	C_22_H_34_O_4_	3.74
26.07	Dotriacontane **(17)**	450	C_32_H_66_	1.68
27.91	Dioctyl hexanedioate **(18)**	370	C_22_H_42_O_4_	1.61
28.53	Oleic acid 3-(octadecyloxy) propyl ester **(19)**	592	C_39_H_76_O_3_	0.58
30.74	Bis(2-ethylhexyl) phthalate **(20)**	390	C_24_H_38_O_4_	48.90

### GC-MS analysis

**Fig. 7:**
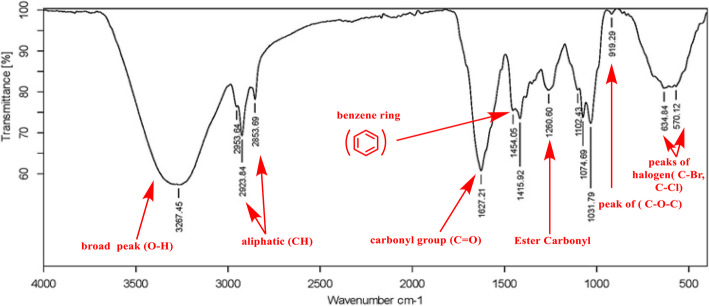
IR spectrum of the EtOAc of NMF6 strain

The chemical composition of the EtOAc of strain NMF6 was evaluated by GC-MS analysis. A total of 20 chemical compounds were identified by comparing their mass spectra with the Wiley Registry8e library based on their retention time, molecular formula, and molecular weight (Fig. 7). As shown in Table 6, > 50% of whole extract represents phthalate esters, diisobutyl phthalate (12) (1.43%), phthalic acid, butyl 8-methylnonyl ester (16) (3.74%), and bis(2-ethylhexyl) phthalate (20) (48.90%), whereas polyketide compounds accounted for approximately 25% of whole extract and miscellaneous compounds.

### Fourier-transform infrared spectroscopy (FTIR)

**Fig. 8:**
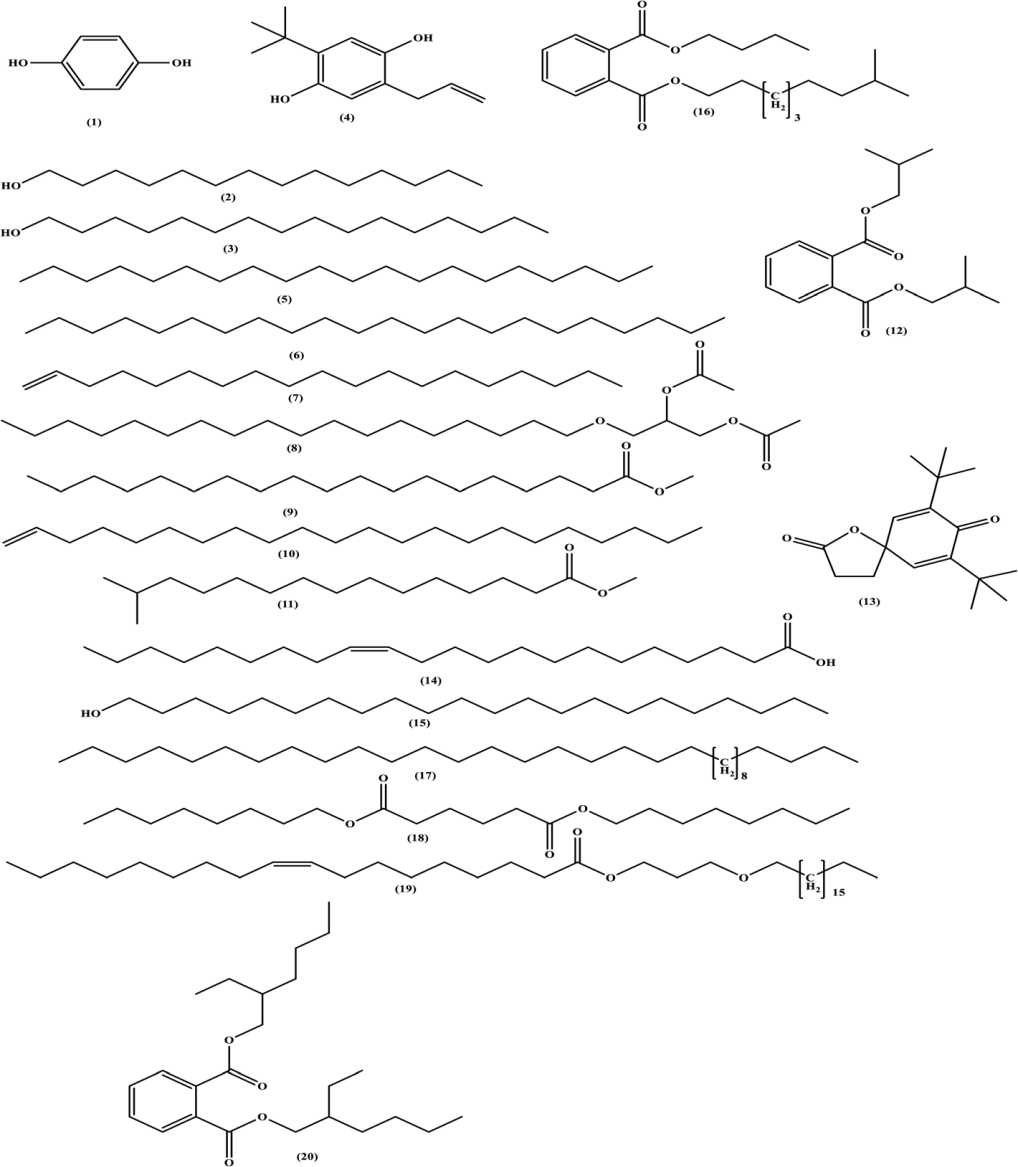
Chemical structures of the compounds identified in NMF6 extract

FTIR analysis of the EtOAc of strain NMF6 revealed the presence of signal at 1454 cm^−1^ that accounted for an aromatic benzene ring, which confirmed the presence of phthalate benzene ring. Moreover, the presence of signal at 1627 cm^−1^ confirmed the presence of carbonyl group **(C=O)** of both aldehyde and ketone, which confirmed the presence of fatty acid and polyketide derivatives. The presence of signals at 2853–2953 cm^−1^ was attributed to the presence of aliphatic **(CH)** group, and finally, the presence of signal at 3267 cm^−1^ accounted for the **(OH)** group (Fig. 8).

## Discussion

We isolated a total of eight actinomycetes associated with the marine sponge *D. ardoukobae* and selected one isolate, coded as strain NMF6, on the basis of the broad-spectrum antimicrobial activity, for further characterization and bioactivity evaluation. The strain was identified as a species belonging to the genus *Streptomyces* and designated as *Streptomyces* sp. NMF6 with the GenBank accession number MW015111. Our results showed that the strain NMF6 produces secondary metabolite(s) exhibiting different bioactivities such as antimicrobial, antioxidant, anticancer, and antiviral effects.

Marine sponges host diverse microbial communities, with biomass accounting for up to 25% of the sponge volume, that produce bioactive compounds formerly attributed to host sponge. Actinomycetes are among the most common phyla associated with marine sponges, with *Streptomyces* being the most abundant genus [13, 40]. Our results showed that *D. ardoukobae* hosts bioactive actinomycetes, with most of them belonging to the genus *Streptomyces*.

The reliable taxonomy of prokaryotes, especially the genus *Streptomyces*, requires data from both DNA-based methods and phenotypic characterization [41]. Several studies have demonstrated that the highly similar *Streptomyces* strain may differ in terms of biochemical profiles and carbon source utilization [42, 43]. Therefore, to provide better understanding and complement the phylogenetic analysis of NMF6 strain, we conducted detailed biochemical and physiological characterization. Our data revealed that the strain NMF6 belonged to the genus *Streptomyces* and could grow at different ranges of NaCl (0–4%, w/v), pH (4–10), and temperature (20–45 °C). These results indicate that the strain is well adapted to the environmental conditions of the mangrove environment, where there are continuous changes in salinity and temperature due to tidal inundation [44, 45].

*Streptomyces* species are well known for the production of secondary metabolites possessing different bioactivities such as antitumor, antiviral, antioxidant, antihypertensive, immunosuppressant, and especially antimicrobial properties, which play a role as defense compounds against microbes competing in the natural environment [46]. The strain NMF6 exhibited antimicrobial activity against members of gram-positive and gram-negative bacteria and yeast.

Free radicals such as reactive oxygen species and reactive nitrogen species are involved in several biological processes in the human body, such as apoptosis, immunity, and cell signaling. Endogenous antioxidative mechanisms, which could involve enzymatic compounds such as superoxide dismutase, catalase (CAT), and glutathione peroxidase or nonenzymatic compounds such as albumin and bilirubin, control the levels of free radicals, as excessive free radicals could damage vital molecules (proteins, lipids, carbohydrates, and DNA) and generate several pathologies. However, under stress conditions, endogenous antioxidative mechanisms became compromised and the free radicals accumulate and result in oxidative stress [47]. The uptake of exogenous antioxidant compounds reduces oxidative stress and prevents several diseases such as cardiovascular disorders, rheumatoid arthritis, ulcerogenesis, acquired immunodeficiency disease, among others. Besides medicinal applications, antioxidants prevent oxidation processes in stored food, especially those containing unsaturated fatty acids [48]. Microorganisms, especially actinomycetes, are a promising source of natural antioxidants that are safer than the hazardous synthetic chemicals [6]. Several studies have reported the antioxidant potential of different *Streptomyces* species isolated from different marine habitats [49–53]. The extract of *Streptomyces* sp. NMF6 demonstrated promising antioxidant activities when tested by the DPPH assay, which implies hydrogen atom transfer, and both phosphomolybdenum and ferric-reducing power assays, which imply single electron transfer (SET). These results indicate that the extract contains bioactive compound(s) exhibiting antioxidant activity through different mechanisms. To evaluate the antioxidant potential of natural extracts and obtain a better understanding of their activities, it is necessary to apply several assays indicating different mechanisms [51, 53], and the results should be expressed as equivalent of a standard antioxidant to allow comparison between the results of different studies and avoid misinterpretation due to variation in applying the methods by different research groups [54]. Therefore, in the present study, we evaluated the antioxidant capacity of *Streptomyces* sp. NMF6 extract using different assays and expressed the results as ascorbic acid equivalents.

Cancer is one of the most fatal diseases worldwide. In 2018, the International Agency for Research on Cancer reported approximately 10 million deaths among 18 million cancer-diagnosed patients in 185 countries [3]. The search for novel anticancer compounds from natural sources is crucial to overcome the phenomenon of drug resistance and the harmful side effects associated with some verified synthetic anticancer drugs [55]. Therefore, researchers investigating natural products have conducted considerable work, especially on marine resources, to fulfill the urgent need for new anticancer drugs with novel modes of action and fewer side effects. Natural products obtained from marine microbes have been confirmed to possess promising anticancer activity in in vitro and in vivo models against various tumor cell lines with different mechanisms of action [56]. Among microorganisms, the genus *Streptomyces* has been used to produce several verified anticancer compounds such as bleomycin, dactinomycin, mitomycin, and doxorubicin [57, 58]. Moreover, several studies have reported the anticancer activity of either pure compounds or crude extracts derived from various marine *Streptomyces* species [7]. In the present study, the crude extract of *Streptomyces* sp. NMF6 associated with *D. ardoukobae* exhibited in vitro anticancer activity against three human cancer cell lines, viz., breast cancer cell line (MCF-7), hepatocellular carcinoma cell line (Hep-G2), and human colon carcinoma cell line (HCT-116), with the IC_50_ values being 55.65, 59.39, and 49.85 μg/mL, respectively. The SI values of *Streptomyces* sp. NMF6 extract for all the tested cancer cell lines were < 2, which indicates limited selectivity [59]. However, the purified compound responsible for the cytotoxic activity could be more selective toward malignant cells.

Virus diseases such as influenza, herpes simplex virus (HSV), human immunodeficiency virus, respiratory syncytial virus, enterovirus 71 (EV71), dengue virus, and Ebola virus, as well as the recently emerging coronavirus disease 2019 (COVID-19), have been a serious threat for human health and have caused millions of human deaths worldwide [60]. As viruses mutate their genome easily and spread rapidly due to urbanization, global travel, and migration, the treatment of viral infections (especially in the absence of vaccines and efficient antiviral drugs) represents a huge challenge [61]. Due to viral resistance and the toxic side effects associated with the existing antiviral drugs, it has become necessary to explore novel antiviral compounds with different mechanisms of action to manage viral infections [62]. Marine microorganisms are well-known producers of antiviral agents. A total of 89 antiviral compounds representing eight structural classes have been isolated from marine microbes between 2015 and 2019 [63]. Several studies have reported that marine streptomycetes are excellent producers of antiviral compounds [64–69]. In the present study, the antiviral activity of EtOAc derived from *Streptomyces* sp. NMF6 was evaluated using the MTT antiviral protocol, and the results showed that the extract exhibited promising antiviral activity against HSV-1, CoxB4, and HAV at nontoxic concentrations, with high SI values of 13.25, 9.42, and 8.25, respectively, which indicate differential selectivity [70]. As viruses are abundant in the marine environment and interact with microbial communities as well as marine organisms [71], the strain NMF6 may produce antiviral secondary metabolites as defensive compounds in response to viral attack of the microbial community or the host sponge.

GC-MS analysis has played a role in the bioprospecting of natural products from streptomycetes [72–74]. More than 50% of the EtOAc of strain NMF6 contained phthalate esters, whereas polyketide compounds accounted for approximately 25% of whole extract. Phthalate ester compounds were earlier known as pollutants, but in the past few years, it has been found that microorganisms such as bacteria and fungi are able to synthesize these compounds [75]. Bis-(2-ethylhexyl) phthalate is the major compound of NMF6 strain extract; this compound has been frequently isolated from several microbial sources such as *Streptomyces* [76], *Nocardia* [77], and *Aspergillus* [78]. Polyketide secondary metabolites are found in bacteria, fungi, and plants and represent one of the largest groups of natural products [79]. They are structurally classified into four groups, viz., aromatics, macrolides, polyethers, and polyenes. Most of these compounds are valuable antibiotics or exhibit other pharmacological activities [80].

## Conclusions

The study has demonstrated that the marine sponge *D. ardoukobae* hosts bioactive actinomycetes, and the potential of *Streptomyces* sp. NMF6 strain as a source of industrial and pharmaceutical products was evaluated. The strain secretes extracellular hydrolytic enzymes of industrial interest and produces secondary metabolites possessing various biological activities. The EtOAc of strain NMF6 exhibited antimicrobial activity against gram-negative and gram-positive bacteria and unicellular fungi; anticancer activity against breast cancer cell line (MCF-7), hepatocellular carcinoma cell line (Hep-G2), and human colon carcinoma cell line (HCT-116); and antiviral activity against HSV-1, HAV, and CoxB4. Furthermore, the extract demonstrated promising antioxidant activities as evaluated by different methods, including DPPH, phosphomolybdenum, and ferric-reducing power assays. Therefore, the strain NMF6 attracts interest for further studies concerning the manipulation of culture conditions to obtain maximum efficiency in producing enzymes and bioactive secondary metabolites. Further studies regarding separation, purification, and structure elucidation of the bioactive compounds are required.

## Data Availability

All data generated or analyzed during this study are included in this published article.

## Abbreviations

EtOAc: Ethyl acetate
TBHQ: Tert-butylated hydroquinone
BHA: Butylated hydro anisole
BHT: Butylated hydro toluene
SCNA: Starch casein nitrate agar
DMSO: Dimethyl sulfoxide
DPPH: 1-Diphenyl-2-picrylhydrazyl
AAEAC: Ascorbic acid equivalent antioxidant capacity
RPMI: Roswell Park Memorial Institute 1640 medium
MTT: 3-(4,5-Dimethylthiazol-2-yl)-2,5-diphenyltetrazolium bromide
IC_50_: Half maximal inhibitory concentration
CC_50_: The concentration that caused 50% toxicity
S: *Staphylococcus*
C: *Candida*
V: *Vibrio*
E: *Enterococcus*
